# Middle and Distal Common Carotid Artery Stenting: Long-Term Patency Rates and Risk Factors for In-Stent Restenosis

**DOI:** 10.1007/s00270-020-02522-5

**Published:** 2020-05-21

**Authors:** Miklós Vértes, Dat T. Nguyen, György Székely, Ákos Bérczi, Edit Dósa

**Affiliations:** Heart and Vascular Center, Semmelweis University, Városmajor Street 68, Budapest, 1122 Hungary

**Keywords:** Carotid artery, Stenting, In-stent restenosis, Hyperlipidemia, Stent fracture

## Abstract

**Purpose:**

In the absence of literature data, we aimed to determine the long-term patency rates of middle/distal common carotid artery (CCA) stenting and to investigate predisposing factors in the development of in-stent restenosis (ISR).

**Materials and Methods:**

Fifty-one patients (30 males, median age 63.5 years), who underwent stenting with 51 self-expandable stents for significant (≥ 60%) stenosis of the middle/distal CCA, were analyzed retrospectively. Patient (atherosclerotic risk factors, comorbidities, medications), vessel (elongation), lesion (stenosis grade, length, calcification, location), and stent characteristics (material, diameter, length, fracture) were examined. Duplex ultrasonography was used to monitor stent patency. The Mann–Whitney *U* and Fisher’s exact tests, Kaplan–Meier analyses, and a log-rank test were used statistically.

**Results:**

The median follow-up time was 35 months (interquartile range, 20–102 months). Significant (≥ 70%) ISR developed in 14 patients (27.5%; stenosis, *N* = 10; entire CCA occlusion, *N* = 4). Primary patency rates were 98%, 92%, 83%, 73%, and 61% at 6, 12, 24, 60, and 96 months, respectively. Reintervention was performed in six patients (11.8%) with nonocclusive ISR. Secondary patency rates were 100% at 6 and 12 months and 96% at 24, 60, and 96 months. In-stent restenosis developed more frequently (*P* < .001) in patients with hyperlipidemia; primary patency rates were also significantly worse (Chi-square, 11.08; degrees of freedom, 1; *P* < .001) in patients with hyperlipidemia compared to those without.

**Conclusion:**

Stenting of the middle/distal CCA can be performed with acceptable patency rates. If intervention is unequivocally needed, patients with hyperlipidemia will require closer follow-up care.

**Level of Evidence:**

Level 3, Local non-random sample.

## Introduction

Compared to other locations [carotid bulb, carotid bifurcation, proximal common carotid artery (CCA)], atherosclerotic stenosis rarely occurs in the middle/distal CCA [1]. Neurological symptoms of CCA stenosis caused by hemodynamic insufficiency or distal embolization [2] can be as severe as those of internal carotid artery (ICA) stenosis and may lead to disability and socioeconomic burden [3]. The economic burden is due to direct health care costs, informal care costs, and indirect costs (e.g. social benefit payments and lost income) [4].

Therapeutic options for significant carotid stenosis include best medical treatment (BMT), endovascular intervention, and surgical reconstruction [5–7]. In contrast to proximal CCA stenosis, none of the guidelines provide any recommendation on the type of invasive therapy for middle/distal CCA stenosis [5–7]. In our institution, the indication of invasive therapy for middle/distal CCA stenosis is the same as that for the proximal CCA, and percutaneous antegrade stenting is the preferred method.

We were unable to identify publications on the long-term patency of middle/distal CCA stenting and the risk factors for in-stent restenosis (ISR). Most studies focused on proximal CCA stenting and found low ISR rates (0–19%), with no predictors of ISR [8–14].

In light of the literature, we aimed to investigate the long-term patency rates of middle/distal CCA stenting and the risk factors for ISR.

## Materials and Methods

### Patients

Our study was based on 51 patients in our department who underwent stenting for significant stenosis of the middle and/or distal third of the CCA between 2000 and 2018. The middle/distal CCA was defined as the segment from 30 mm cranial on the left side and 15 mm cranial on the right side to the CCA origin to 10 mm caudal to the carotid bifurcation.

Institutional Review Board approval was granted, and informed consent was obtained from all patients who had fluoroscopy for evaluation of stent fracture (SF).

### Preprocedural Workup and Stenting Protocol

Diagnosis of middle/distal CCA stenosis was established with duplex ultrasonography (DUS), computed tomography angiography (CTA), or magnetic resonance angiography, and was verified with digital subtraction angiography (DSA) during the procedure. The preprocedural workup and stenting protocol have been described previously [14]. The indication for intervention was the presence of either asymptomatic but ≥ 70% luminal narrowing (*N* = 23 [45.1%]) or symptomatic and ≥ 60% stenosis (*N* = 28 [54.9%]). Asymptomatic patients underwent stenting if showing multivessel supra-aortic steno-occlusive disease (Table 1). Also, all asymptomatic patients were thought to have an increased risk for stroke even while on BMT. Patients who had episodes of neurological dysfunction caused by focal carotid territory brain or retinal ischemia within the preceding 6 months were defined as symptomatic [15]. In patients with suspected transient ischemic attack (TIA) or acute stroke, urgent brain CT was performed. Patients were scheduled for stenting within 14 days of the onset of an ischemic neurological event. Treatment decisions were made by our vascular team, which included interventional radiologists, vascular surgeons, and consulting neurologists.

**Table 1:** Indications for the treatment of asymptomatic middle/distal common carotid artery stenosis

Indications, *N* (%)	Patients (*N* = 23)
Left middle/distal CCA + contralateral ICA significant stenosis	2 (8.7)
Left middle/distal CCA significant stenosis + contralateral ICA occlusion	7 (30.4)
Left middle/distal CCA + ipsilateral VA significant stenosis	1 (4.3)
Left middle/distal CCA + ipsilateral proximal SA significant stenosis	1 (4.3)
Left middle/distal CCA significant stenosis + ipsilateral proximal SA occlusion	3 (13)
Left middle/distal CCA significant stenosis + contralateral TIA/stroke	5 (21.7)
Right middle/distal CCA + contralateral ICA significant stenosis	2 (8.7)
Right middle/distal CCA significant stenosis + contralateral ICA occlusion	1 (4.3)
Right middle/distal CCA + ipsilateral proximal SA significant stenosis	1 (4.3)

*CCA* Common carotid artery, *ICA* internal carotid artery, *SA* subclavian artery, *TIA* transient ischemic attack, *VA* vertebral artery

**Fig. 1:**
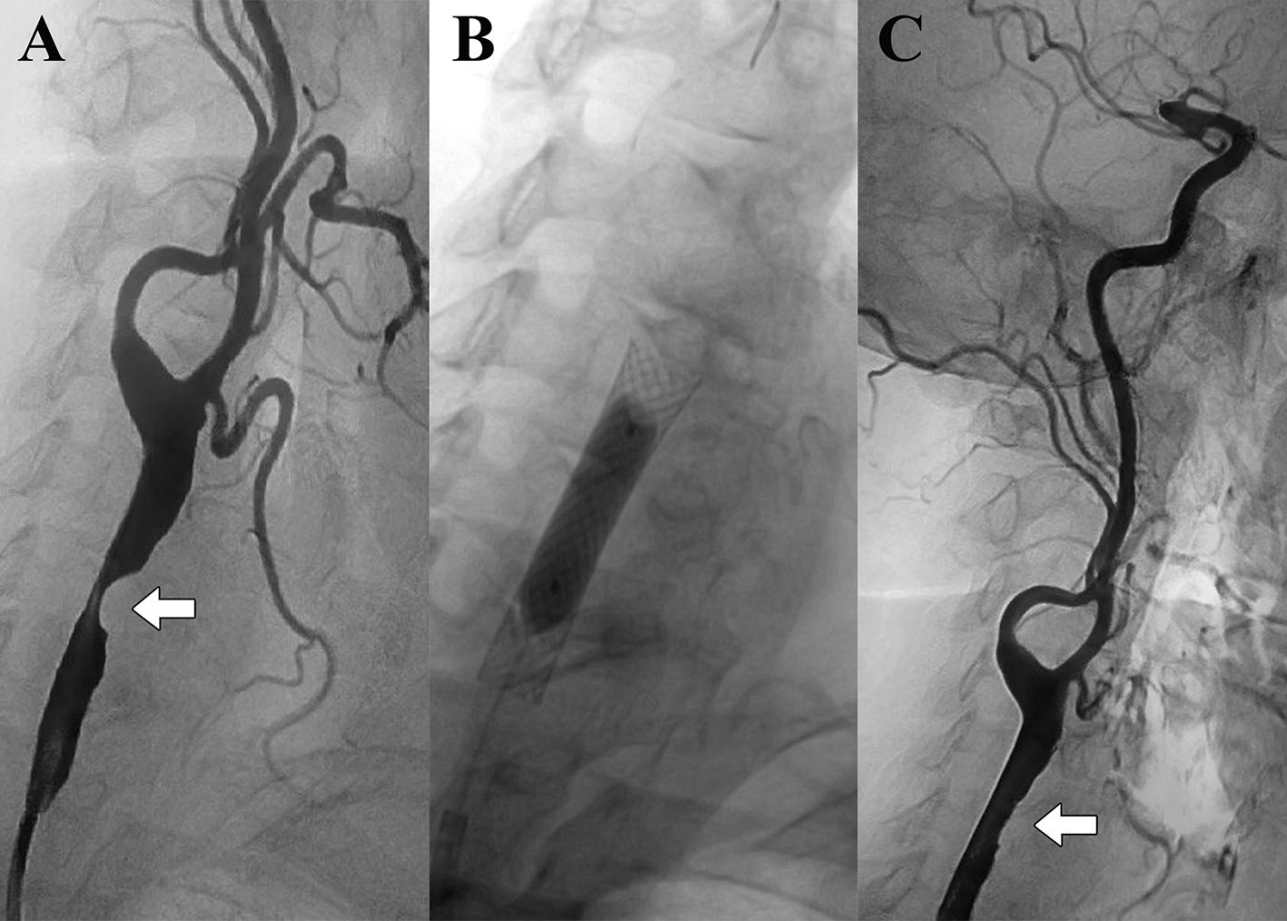
An example of distal common carotid artery stenting **A**. Digital subtraction angiography image showing high-grade stenosis in the distal part of the right common carotid artery. **B**. After implantation of a Wallstent (7 × 30 mm), postdilation was performed with a Sterling balloon (6 × 20 mm). **C**. Minimal residual stenosis can be seen on the completion angiogram.

Self-expandable stents were implanted in all patients; predilation was carried out in only two cases, but postdilation was routinely performed (Table 2, Fig. 1). The use of a cerebral protection device (FilterWire EZ, Boston Scientific Corp., Marlborough, MA, USA) was left to the discretion of the interventional radiologist and was applied in 40 cases (78.4%). Technical success was defined as ≤ 30% residual stenosis. Dual antiplatelet therapy was started at least 3 days prior to intervention (or as a single loading dose in urgent cases) and lasted for 1–3 months; after that, if there were no other indications, only single antiplatelet regimen was recommended.

**Table 2:** Parameters of the stents and balloons

Stents/balloons	Manufacturer	Size (mm), diameter × length
Self-expandable stents (*N* = 51)		
Wallstent (*N* = 39)	Boston Scientific Corp., Marlborough, MA, USA	7–10 × 20–50
Precise Pro (*N* = 5)	Cordis Corp., Johnson & Johnson Co., Miami, FL, USA	8–10 × 40
S.M.A.R.T. Control (*N* = 4)	Cordis Corp., Johnson & Johnson Co., Miami, FL, USA	8–10 × 40–80
Epic (*N* = 1)	Boston Scientific Corp., Marlborough, MA, USA	10 × 100
Exact (*N* = 1)	Abbott Vascular Inc., Santa Clara, CA, USA	9 × 30
Nexstent (*N* = 1)	Boston Scientific Corp., Marlborough, MA, USA	9 × 30
Balloons used for predilation (*N* = 2)		
Emerge (*N* = 1)	Boston Scientific Corp., Marlborough, MA, USA	4 × 30
Sprinter legend RX (*N* = 1)	Medtronic Inc., Minneapolis, MN, USA	4 × 20
Balloons used for postdilation (*N* = 51)		
Ultra-soft SV (*N* = 19)	Boston Scientific Corp., Marlborough, MA, USA	7 × 20
Sterling (*N* = 17)	Boston Scientific Corp., Marlborough, MA, USA	6–8 × 20–40
Rx Viatrac 14 Plus (*N* = 10)	Abbott Vascular Inc., Santa Clara, CA, USA	6–7 × 20–40
Maverick (*N* = 5)	Boston Scientific Corp., Marlborough, MA, USA	6 × 20

### Follow-up

Follow-up examinations, which were scheduled at 6 weeks, 6 months, 12 months, and then yearly after the stenting, included review of the medical records of the patient, a basic neurological evaluation, and assessment by DUS of the neck arteries on both sides. In patients with abnormal DUS (direct sign: ≥ 300 cm/s peak systolic velocity within or at the ends of the CCA stent [7]; indirect sign: tardus-parvus waveform in the ICA [9]), significant (≥ 70%) ISR was suspected. Stent occlusion was diagnosed when neither color nor Doppler signal was detected in the stent. The presence of significant ISR/stent occlusion was confirmed by CTA or DSA.

Primary patency was defined as stents without significant ISR. Secondary patency was defined as open stents after endovascular reintervention due to ISR.

### Analyzed Parameters

Patients were divided into two groups based on the presence or absence of significant ISR.

1. Patient data: atherosclerotic risk factors (age, gender, smoking, hypertension, hyperlipidemia, diabetes mellitus, and obesity) and medication regimen (antiplatelet, lipid-lowering, and cilostazol therapies). Hypertension, hyperlipidemia, and diabetes mellitus were assumed to be present if they were noted in the medical reports of the patient, and/or if the patient was taking drugs for the disease or was on insulin therapy for diabetes mellitus. A body mass index of ≥ 30 kg/m^2^ was defined as obesity [16].
2. Vessel-related parameter: elongation of the CCA. Elongation was defined as an S- or C-shaped tortuosity or undulation [17].
3. Lesion-related parameters: degree and length of the stenosis, presence and grade of calcification, and location of the lesion (left or right side, middle and/or distal third). The grade and length of stenosis were assessed on DSA images, as described by Bonati et al. [18], while the presence and grade of calcification were judged on fluoroscopic images, as reported by Doris et al. [19].
4. Stent characteristics: material, diameter, length, and fracture. During follow-up in 2018, patients were asked to return for a fluoroscopic examination of the implanted stents to determine the presence of SF; fractures were categorized according to a classification proposed by Nakazawa et al. [20]. For details of evaluation of SF, please see a publication by Hüttl et al. [21].

### Statistical Analysis

Statistics were calculated using StatSoft Statistica 13.4 (Moonsoft Oy, Espoo, Finland) and GraphPad Prism 7.01 (GraphPad Software Inc., La Jolla, CA, USA) software. Continuous data were presented as medians and interquartile ranges (IQR: Q1, Q3); categorical data were given as counts (percentages). The relationship between ISR and other variables was evaluated with the Mann–Whitney *U* test for continuous data and Fisher’s exact test for categorical data. A Kaplan–Meier analysis was performed to determine primary and secondary patency rates. Patients were dichotomized based on the presence/absence of the only variable where *P* < .05, and Kaplan–Meier curves of the resulting subsamples were compared with a log-rank test. All statistical tests were two-tailed. The threshold of statistical significance was *P* < .05.

## Results

### Patient, Vessel, Lesion, and Stent Data

A total of 68 patients were treated for steno-occlusive disease of the middle/distal CCA during the examined period. Those patients, who had anamnestic history of prior ipsilateral carotid surgery (*N* = 7) or irradiation in the neck region (*N* = 7), or in whom the angiographic or DUS morphology was highly suspicious of carotid fibromuscular dysplasia (*N* = 2) or arteritis (*N* = 1), were excluded from the study. In the remaining 51 patients, who underwent radiological intervention with 51 self-expandable stents (Table 2), atherosclerosis was the putative etiology of the stenosis. Patient-, vessel-, lesion-, and stent-related parameters are summarized in Table 3.

**Table 3:** Patient-, vessel-, lesion-, and stent-related parameters

Characteristics	All patients (*N* = 51)	ISR group (*N* = 14)	Non-ISR group (*N* = 37)	*P* value
*Patient-related parameters*				
Atherosclerotic risk factors				
Age (year), median (IQR)	63.5 (55.2–68.3)	64.2 (58.3–66.7)	62.7 (55.2–68.7)	.908
Female sex, *N* (%)	21 (41.2)	8 (57.1)	13 (35.1)	.206
Smoking (current or former), *N* (%)	46 (90.2%)	12 (85.7)	34 (91.9)	.606
Hypertension, *N* (%)	50 (98%)	14 (100)	36 (97.3)	> .999
Hyperlipidemia, *N* (%)	33 (64.7%)	14 (100)	19 (51.4)	< .001
Diabetes mellitus, *N* (%)	17 (33.3%)	6 (42.9)	11 (29.7)	.507
BMI (kg/m^2^), median (IQR)	26.3 (23.2–29.4)	23.5 (22–27.9)	26.7 (24.2–29.4)	.351
Obesity (BMI ≥ 30 kg/m^2^), *N* (%)	11 (21.6%)	3 (21.4)	8 (21.6)	> .999
Medication regimen				
Moderate- or high-intensity statin therapy, *N* (%)	38 (74.5)	13 (92.9)	25 (67.6)	.081
Other lipid-lowering medication, *N* (%)	1 (2)	1 (7.1)	0 (0)	.274
Acetylsalicylic acid therapy, *N* (%)	15 (29.4)	4 (28.6)	11 (29.7)	> .999
Clopidogrel therapy, *N* (%)	12 (23.5)	2 (14.3)	10 (27)	.471
Dual antiplatelet therapy for 1–3 months, *N* (%)	51 (100)	14 (100)	37 (100)	> .999
Long-term dual antiplatelet therapy, *N* (%)	24 (47.1)	8 (57.1)	16 (43.2)	.531
Cilostazol therapy, *N* (%)	6 (11.8)	2 (14.3)	4 (10.8)	.661
*Vessel-related parameter*				
Elongated CCA, *N* (%)	4 (7.8)	2 (14.3)	2 (5.4)	.300
*Lesion-related parameters*				
Stenosis grade (%), median (IQR)	80 (75–90)	80 (75–90)	80 (75–90)	.319
Length (mm), median (IQR)	13 (10–20)	14 (10–18)	12 (9–20)	.668
Calcification, *N* (%)	11 (21.6%)	3 (21.4)	8 (21.6)	> .999
Heavy calcification, *N* (%)	6 (11.8)	1 (7.1)	5 (13.5)	> .999
Location				
Left side, *N* (%)	37 (72.5%)	12 (85.7)	25 (67.6)	.296
Isolated middle segment, *N* (%)	26 (51)	10 (71.4)	16 (43.2)	.116
Isolated distal segment, *N* (%)	22 (43.1)	4 (28.6)	18 (48.6)	.224
Middle and distal segments, *N* (%)	3 (5.9)	0 (0)	3 (8.1)	.552
*Stent-related parameters*				
Material				
Elgiloy self-expandable, *N* (%)	39 (76.5)	13 (92.9)	26 (70.3)	.141
Diameter (mm), median (IQR)	8 (7–9)	7 (7–9)	8 (7–9)	.227
Length (mm), median (IQR)	30 (30–40)	30 (30–40)	40 (30–40)	.280
Fracture,^a^*N* (%)	2 (4.3)	0 (0)	2 (5.9)	> .999

*BMI* Body mass index, *CCA* common carotid artery, *IQR* interquartile range, *ISR* in-stent restenosis

^a^Stent fracture was examined in 47 patients (ISR group, *N* = 13; non-ISR group, *N* = 34)

### Early Postprocedural Period (≤ 30 days)

Technical success was achieved in all patients. The following four complications (7.8%) were observed: one femoral pseudoaneurysm, which was eliminated by ultrasound-guided injection of thrombin; and one allergic reaction to contrast material causing perioral edema and urticaria, which was treated with chloropyramine and methylprednisolone. Two neurological complications developed: one contralateral hemiparesis plus aphasia that lasted for 5 min after balloon inflation; and one transient contralateral upper extremity numbness. A cerebral protection device was utilized in both these patients, with debris found in the filter of the latter. All neurological symptoms disappeared spontaneously. Computed tomography examination performed within 2 h of the onset of symptoms revealed no evidence of acute brain ischemia or intracranial arterial obstruction in either patient. The 30-day all-cause mortality rate was zero.

### Follow-up Period

**Fig. 2:**
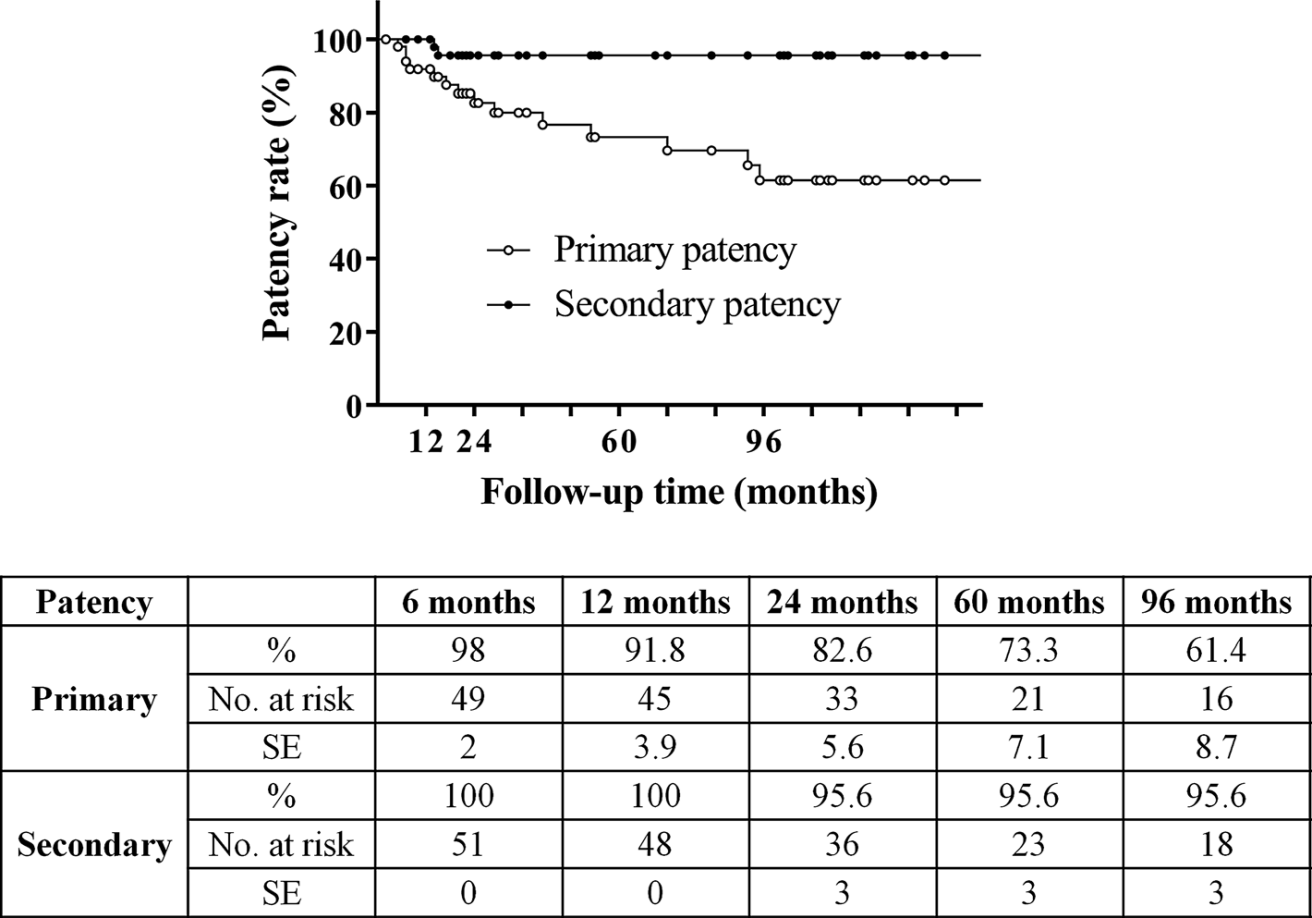
Primary and secondary patency rates *No.* Number, *SE* standard error

The median follow-up time was 35 months (IQR, 20–102 months). Significant (≥ 70%) ISR developed in 14 patients (27.5%; stenosis, *N* = 10; entire CCA occlusion, *N* = 4). Reintervention (percutaneous transluminal angioplasty [PTA] with a plain balloon, *N* = 5; restenting, *N* = 1) was conducted in six patients (11.8%) with nonocclusive ISR; among them, two patients had ipsilateral TIA, while four had rapid ISR progression on BMT. The remaining patients with nonocclusive ISR or entire CCA occlusion were asymptomatic and received BMT. Recurrent ISR was noted in two cases: one was treated with PTA with a drug-eluting balloon (Ranger, 7 × 40 mm, Boston Scientific Corp., Marlborough, MA, USA), while the other continued on BMT. Primary and secondary patency rates are shown in Fig. 2. Ischemic neurological symptoms unrelated to the treated CCA were observed in five patients (9.8%; contralateral TIA, *N* = 2; contralateral minor stroke, *N* = 1; vertebrobasilar events, *N* = 2).

Of 51 patients, 47 (92.2%) returned for a fluoroscopic examination of the implanted stents. Two SFs (4.3%; one class I: fracture of one strut and one class III: fracture of multiple struts with stent deformity) were detected.

### Predictors of In-Stent Restenosis

In-stent restenosis developed more frequently in patients with hyperlipidemia (*P* < .001) (Table 3). All patients with ISR had hyperlipidemia. Other patient-, vessel-, lesion-, and stent-related parameters, including SF, did not differ significantly between the two groups (Table 3).

**Fig. 3:**
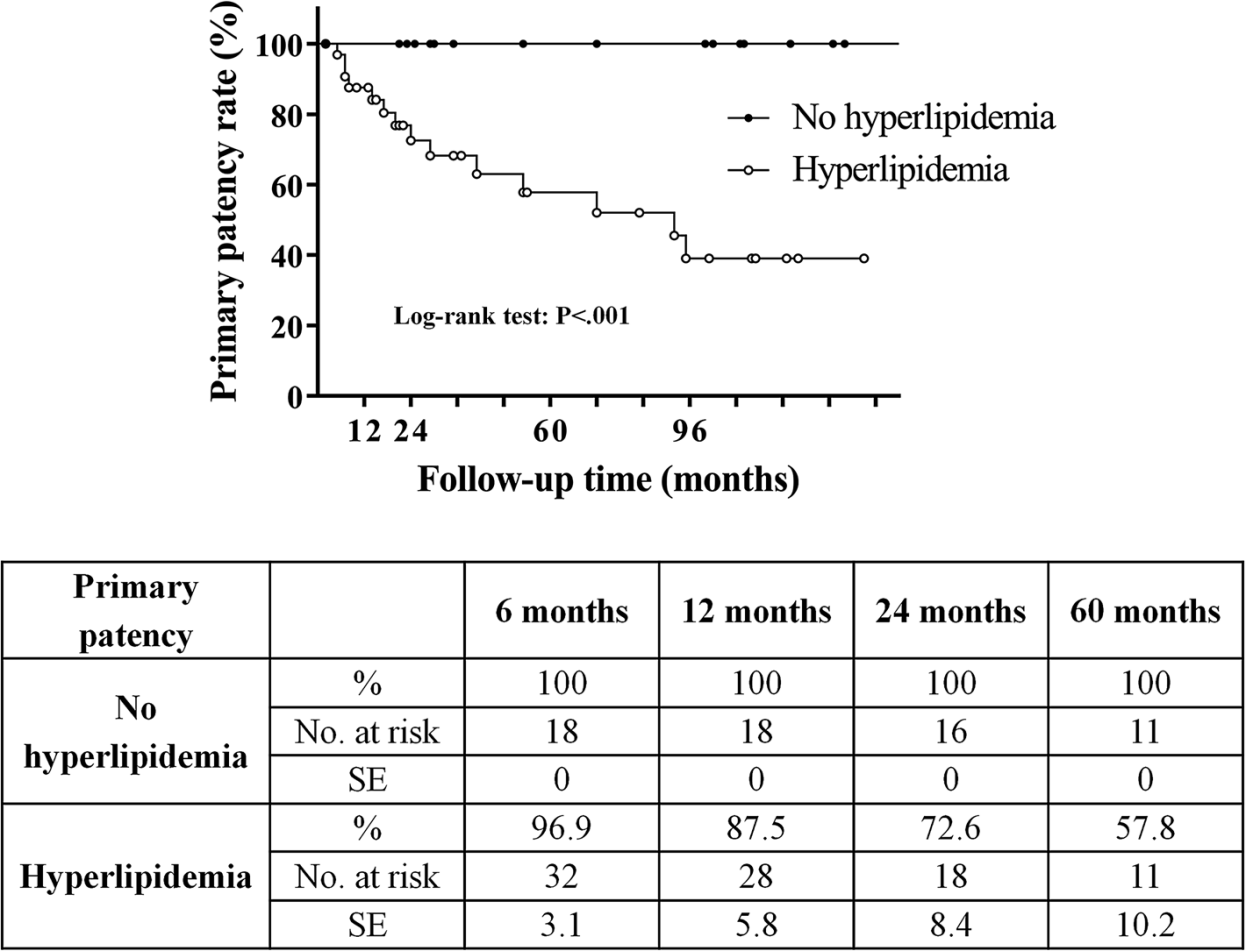
Primary patency rates for patients with and without hyperlipidemia *No.* Number, *SE* standard error

The primary patency rate was 100% at 6, 12, 24, and 60 months in patients without hyperlipidemia, while it was 97%, 88%, 73%, and 58% at 6, 12, 24, and 60 months, respectively, in patients with hyperlipidemia. The primary patency rates were significantly worse (Chi-square, 11.08; degrees of freedom, 1; *P* < .001) in patients with hyperlipidemia compared to those without (Fig. 3).

## Discussion

Similarly to ICA stenosis, invasive therapy for CCA stenosis only in symptomatic and those asymptomatic patients with at least one clinical and/or imaging characteristic (history of contralateral TIA/minor stroke; presence of silent brain infarction; detection of stenosis progression and/or large/vulnerable carotid plaque; evidence of spontaneous embolization on transcranial Doppler monitoring; coexistence of intracranial disease, etc.) that makes them at “higher risk for stroke” on BMT is recommended [5, 7, 22]. The Carotid Revascularization and Medical Management for Asymptomatic Carotid Stenosis Trial (CREST-2) is designed to further refine the treatment of asymptomatic patients with high-grade carotid artery stenosis [23], but its final results are still several years away. Nowadays, in case of proximal CCA stenosis, open retrograde stenting is increasingly frequently applied because it minimizes the chance of intraoperative complications and embolic events during and after the procedure [7, 24]. In contrast to ostial CCA lesions, open retrograde stenting is often technically not feasible in patients with middle/distal CCA stenosis, and the rates of periprocedural stroke and mortality of the open surgical reconstructions are not negligible (1–8% and 0.4–8%, respectively [25–29]); therefore, in our institute, percutaneous antegrade stenting has become the first treatment of choice for middle/distal CCA steno-occlusive disease.

The technical success rate of percutaneous antegrade stenting of proximal CCA ranges between 95 and 100% [8–14]. Access site complications were observed in less than 6% of patients [8–14]. So far, only one procedure-related death was reported; this was due to retroperitoneal bleeding [8–14]. Transient ischemic attack occurred in 0–5.9% (ipsilateral, 0–2%), ipsilateral minor stroke in 0–4.7%, ipsilateral major stroke in 0–2%, and myocardial infarction in 0–1.5% within 30 days following antegrade stenting of the proximal CCA [8–14]. In a study by Tang et al., 66.7% of symptomatic patients were relieved of initial symptoms, and the rest showed improvement [13]. In the current work, the technical success and complication rates were similar to those mentioned above.

The prevalence of proximal CCA ISR is 0–19% [8–14]. The patency was examined by Paukovits et al., who showed a primary patency rate of 58% at 60 months in patients who underwent percutaneous antegrade proximal CCA stenting [11]. Our study revealed significant ISR in 27.5% of patients and a 73% primary patency rate at 60 months. Our 27.5% ISR rate is worse than those noted in proximal CCA [8–14]. No definite explanation can be given for our higher ISR rate, but differences in patient, lesion, and stent characteristics among studies can be presumed.

No predictors of CCA ISR have been identified to date. Corresponding to other studies [30, 31], we also evaluated several possible risk factors and found hyperlipidemia to be significantly more common among patients with ISR. The role of hyperlipidemia in the formation of neointimal hyperplasia has also been demonstrated by other research groups [32–36]. Hyperlipidemia increases the entry of low-density lipoprotein (LDL) into the intima and its progressive oxidative alteration in the subendothelial space. Oxidized LDL results in further lipid infiltration across the intact endothelium, where it aggregates and activates the release of mitogens from platelets, macrophages, and endothelial cells; this, in turn, stimulates smooth muscle cell proliferation, thereby leading to neointima formation [32, 33].

The middle/distal CCA has not been examined before in the context of SF. The SF rate was reported to be 39% in patients treated for proximal CCA stenosis [14]. In the present study, the SF rate was much lower (4.3%). Stent fractures have several known predictors (stent design and length, grade of residual stenosis, etc.) [14, 37–39], but the two most important ones are the location of the stent and calcification of the lesion [14, 37–39]. On the one hand (in general), the low SF rate in this patient population can be explained by the less significant effect of the beating heart and shear forces from the curvature of the aortic arch compared to the proximal CCA. On the other hand (in the current study), the number of heavily calcified lesions was not deemed to be considerable.

Our results should be regarded in the light of several limitations. First, the study was retrospective in nature. Second, the sample size was small and inhomogeneous, which did not permit detailed regression analyses in terms of risk factors for ISR or stent occlusion. Third, not all patients had CTA preprocedurally; therefore, fluoroscopic images were used to judge the presence and grade of calcification. Fourth, different stents were implanted in the middle/distal CCA.

## Conclusion

Stenting of the middle/distal CCA can be performed with acceptable patency rates. If intervention is unequivocally needed, patients with hyperlipidemia would require closer follow-up care. Further studies with a larger and more homogeneous sample may be necessary to confirm our results and to be able to perform important subgroup analyses.
